# Enhanced Ethanol Production From Carbon Monoxide by Enriched *Clostridium* Bacteria

**DOI:** 10.3389/fmicb.2021.754713

**Published:** 2021-10-28

**Authors:** Yaxue He, Piet N. L. Lens, María C. Veiga, Christian Kennes

**Affiliations:** ^1^Chemical Engineering Laboratory, Faculty of Sciences and Center for Advanced Scientific Research (CICA), BIOENGIN Group, University of A Coruña (UDC), A Coruña, Spain; ^2^National University of Ireland Galway, Galway, Ireland

**Keywords:** carbon monoxide, *Clostridium* spp., ethanol, butanol, fermentation

## Abstract

Carbon monoxide (CO)-metabolizing *Clostridium* spp. were enriched from the biomass of a butanol-producing reactor. After six successive biomass transfers, ethanol production reached as much as 11.8 g/L with minor accumulation of acetic acid, under intermittent gas feeding conditions and over a wide pH range of 6.45–4.95. The molar ratio of ethanol to acetic acid exceeded 1.7 after the lag phase of 11 days and reached its highest value of 8.6 during the fermentation process after 25 days. Although butanol production was not significantly enhanced in the enrichment, the biomass was able to convert exogenous butyric acid (3.2 g/L) into butanol with nearly 100% conversion efficiency using CO as reducing power. This suggested that inhibition of butanol production from CO was caused by the lack of natural butyric acid production, expectedly induced by unsuitable pH values due to initial acidification resulting from the acetic acid production. The enriched *Clostridium* population also converted glucose to formic, acetic, propionic, and butyric acids in batch tests with daily pH adjustment to pH 6.0. The *Clostridium* genus was enriched with its relative abundance significantly increasing from 7% in the inoculum to 94% after five successive enrichment steps. Unidentified *Clostridium* species showed a very high relative abundance, reaching 73% of the *Clostridium* genus in the enriched sludge (6th transfer).

## Graphical Abstract

Enrichment of *Clostridium* bacteria from carbon monoxide for enhanced ethanol production.

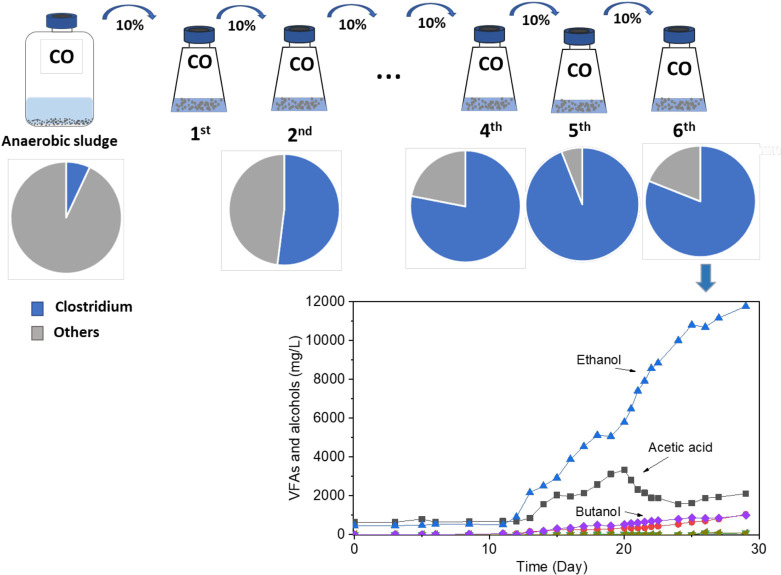

## HIGHLIGHTS

- CO metabolizing acetogens were enriched for selective ethanol production.

- 11.8 g/L ethanol was produced with only minor acetic acid accumulation.

- The enriched acetogens converted 100% exogenous butyric acid to butanol.

- *Clostridium* genus was enriched from 7 to 94% abundance, with 74% unidentified species.

## Introduction

A significant portion of biomass sources like straw and wood is poorly degradable, but the gasification of these carbon-rich waste materials to produce synthesis gas mainly with carbon monoxide (CO) and H_2_ as a starting substrate for fermentation could offer a solution to this problem (Mohammadi et al., 2011). CO is a toxic gas and is present in several industrial gaseous emissions such as those of steel plants (Yu et al., 2015). Its microbiological conversion to (bio)fuels such as ethanol and butanol *via* the water–gas shift reaction by microorganisms has gained increasing attention recently (Fernández-Naveira et al., 2017c).

The low energy density and toxicity of CO limits its application in environmental biotechnology, but a limited number of acetogens can convert CO to acids, ethanol, and, occasionally, butanol and even hexanol (Fernández-Naveira et al., 2017c). These acetogens possess the key CO dehydrogenase enzyme, which converts CO to CO_2_, with acetyl-CoA as the main intermediate, following the Wood–Ljungdahl pathway (WLP) (Fernández-Naveira et al., 2017c). The production of alcohols from CO takes place in two stages, i.e., first, accumulation of volatile fatty acids takes place, and then solventogenesis occurs (Richter et al., 2013; Abubackar et al., 2018). Only a low number of solventogenic carboxydotrophic acetogens have been isolated so far from a variety of environments such as soil, sediments, anaerobic sludge, and animal manure, including *Clostridium ljungdahlii* (Tanner et al., 1993), *Clostridium autoethanogenum* (Abrini et al., 1994), *Clostridium carboxidivorans* (Liou et al., 2005), and *Butyribacterium methylotrophicum* (Lynd et al., 1982). Other acetogenic bacteria, known to produce acetic acid, have only recently been shown to have solventogenic potential with the accumulation of high amounts of ethanol, including *Clostridium aceticum* (Arslan et al., 2019), *Acetobacterium wieringae* strain JM grown on 1.70 bar CO (Arantes et al., 2020), and *Clostridium* sp. AWRP grown on syngas (Lee et al., 2019).

Although syngas bioconversion has been studied with several pure strains, mixed culture fermentations may be easier to implement at large scale than pure cultures, as they may be more resistant and do not require sterile conditions (Charubin and Papoutsakis, 2019). More importantly, the presence of a broad range of acetogenic organisms in mixed cultures could have the potential to achieve metabolic yields that are theoretically not possible in pure cultures. Although mixed cultures may raise challenges of stability of the microbial composition, these can be overcome by controlling parameters such as pH and CO substrate concentration. Mixed cultures may exhibit syntrophic or complementary behavior and may thus better withstand poor environmental conditions such as a low pH or nutrient limitation and have better abilities for adaptation. For instance, *C. autoethanogenum* can convert CO or syngas to ethanol and acetate, but when co-cultured with *Clostridium kluyveri*, the co-culture ends up producing butanol or hexanol with CO as reducing power, not found in any of those individual strains (Diender et al., 2016).

Despite this huge potential, hardly any study has reported and optimized ethanol and butanol production in mixed cultures using 100% CO as the carbon source. Chakraborty et al. (2019) investigated a two-stage fermentation using anaerobic sludge as inoculum, in which first, 6.18 g/L acetic acid was produced from continuous CO gas feeding by an enriched anaerobic sludge at a controlled pH of 6.2. Then, in the same continuous stirred tank reactor (CSTR), 11.1 g/L ethanol and 1.8 g/L butanol accumulated when the pH was decreased to 4.9. The aim of this study was to first enrich CO-metabolizing, solvent-producing acetogens using 100% CO as the carbon and energy source. The enriched bacteria were then determined, and the functional acetogens were identified *via* microbial community analysis. Second, this study further explored the effect of exogenous butyric acid and glucose on enhanced butanol production and also the effect of pH regulation on enhanced ethanol and butanol production by the enriched acetogenic bacteria.

## Materials and Methods

### Source of Inoculum

The inoculum was obtained from a fed batch incubation producing for 6.8 g/L butanol operated with intermittent gas feeding using CO as the sole carbon and energy source after 127 days of operation (He et al., 2022). The microbial community of the inoculum was mainly composed of Clostridia (37%) and Bacilli (36%) classes (He et al., 2022).

### Medium Composition

The culture medium was prepared as described previously (He et al., 2021). A 1 L culture medium was prepared according to Stams et al. (1993) and modified as follows: 408 mg/L KH_2_PO_4_, 534 mg/L Na_2_HPO_4_⋅2H_2_O, 300 mg/L NH_4_Cl, 300 mg/L NaCl, 100 mg/L MgCl_2_⋅6H_2_O, 110 mg/L CaCl_2_⋅2H_2_O, 1 ml trace metal, and 1 ml vitamin stock solution (Stams et al., 1993). Once prepared, the 1 L medium (except for CaCl_2_⋅2H_2_O and vitamins) was brought to boiling in order to remove O_2_, and it was later cooled down to room temperature under an oxygen-free N_2_ flow. Then, CaCl_2_⋅2H_2_O and the vitamins were added, as well as Na_2_S (0.24 g) as the reducing agent.

### Experimental Set-Up

#### Enrichment of Carbon Monoxide-Converting Acetogens

Enrichments were obtained by successive transfer of active cultures (10% v/v) into fresh medium with a headspace CO pressure of 1.8 bar. A 100 ml medium was dispensed into 500 ml flasks, 10 ml enriched sludge was added as inoculum, and the pH was adjusted to 6.2 with 2 M HCl under CO gas flow. The bottles were then sealed with rubber stoppers, capped with screw caps, and incubated with steady agitation (150 rpm) in the dark. When the gas pressure decreased below 1 bar, as a result of bacterial C_1_-gas consumption, the bottle was flushed with fresh pure CO for approximately 5 min, until again reaching a gas pressure of 1.8 bar. To avoid inhibition of solventogenesis at low pH, its value was adjusted to 6.0–6.4 at the beginning of each CO addition. To enhance cell growth, yeast extract was added, after filtration through a 0.22 μm filter, to reach a final concentration of 0.5 g/L. As soon as microbial growth was observed, i.e., OD_600_ increased and acetic acid and alcohols were produced, 10 ml inoculum was transferred into another flask under the same conditions.

In the 6th transfer, the last CO addition started with an initial CO gas pressure of only 1.5 bar, as the glass serum bottles started after 27 days of fermentation under gas pressures as high as 1.8 bar. In the 7th and 8th transfers, the CO pressure was further decreased to 1.2 bar.

#### Enhanced Butanol Production From Exogenous Butyric Acid by Enriched *Clostridium* Populations

To assess and demonstrate the butanol production potential of the enriched culture after the 5th transfer on CO, the enriched sludge was further inoculated in a 1 L serum bottle with 200 ml culture medium and with the addition of 3.2 g/L exogenous butyric acid. The initial pH was 6.2, and 100% CO was introduced as electron donor to reach an initial gas pressure of 1.8 bar. The same CO feeding procedure was used as described in Section “Enrichment of CO Converting Acetogens”.

#### Exogenous Glucose Consumption by the Enriched Sludge

To investigate the sugar utilization and possible endogenous acid production used for ethanol and butanol production, the enriched biomass of the 6th transfer with dominant *Clostridium* spp. was incubated with either pure glucose or glucose and CO as the substrates. A 25 ml medium was dispensed into 125 ml conical flasks, 2.5 ml enriched sludge was added as inoculum (10%), and the pH was adjusted to 6.2 with 2 M HCl under N_2_ gas flow. The headspace was flushed with N_2_ or CO, and glucose was added to reach a final concentration of 5 g/L in duplicate experiments. The bottles were then sealed with rubber stoppers, capped with screw caps, and incubated with steady agitation (150 rpm) in a dark environment.

#### Ethanol Production From Carbon Monoxide With pH Control at 6.2 and 5.7 in Intermittent Gas-Fed Bioreactors

The poor butanol production among the transfers was expected to be due to the low butyric acid production, likely induced by unfavorable pH values and the natural pH drop. Therefore, CO conversion by the enriched culture was further investigated under pH-controlled conditions at pH 5.7 and 6.2, and also without pH control using 10% enriched sludge from the 7th transfer. pH control experiments were carried out in two 1 L serum bottles with 200 ml culture medium and with pH control at either pH 5.7 or 6.2 by supplying 1 M HCl or 1 M NaOH. The same CO feeding procedure was used as described in Section “Enrichment of CO Converting Acetogens”.

#### Sampling

The gas pressure was measured daily. Liquid samples (1 ml) were withdrawn for measuring the cell concentration (OD_600_) and then centrifuged at 8,000× *g* for 5 min, and the supernatant was used to quantify the concentrations of acids and solvents.

### Analytical Methods

The cell concentration was determined with a spectrophotometer (Hitachi, Model U-200, Pacisa & Giralt, Spain) at a wavelength of 600 nm with medium solution as the blank (Arslan et al., 2019). pH was measured by a pH meter (Mettler Toledo, Zurich, Switzerland).

Acetic acid, propionic acid, butyric acid, ethanol, and butanol were determined on a high-performance liquid chromatography (HPLC, HP1100, Agilent Co., United States) equipped with a refractive index detector using an Agilent Hi-Plex H Column (300 × 7.7 mm). A 5 mM H_2_SO_4_ solution was used as mobile phase at a flow rate of 0.80 ml/min, with a sample injection volume of 20 μl and a column temperature of 45°C (Arslan et al., 2019).

### Microbial Analysis

The microbial community composition of different successive transfers of the inoculum, 2nd, 4th, 5th, 6th, 7th, and 8th, exogenous butyric acid addition (HBu), glucose fermentation (Glucose), and pH 6.2 and 5.7 were analyzed. DNA was extracted using a DNeasy^®^ PowerSoil Kit (QIAGEN, Germany) following the manufacturer’s protocol. A 10 ml sludge was used for DNA extraction at the end of the incubations of successive transfers. The extracted DNA was quantified, and its quality was checked by a Nanodrop 2000c Spectrophotometer (Thermo Scientific, United States). The extracted DNA was analyzed by Metagenomics-Seq (Illumina PE150, Q30 ≥ 80%) (Novogene, United Kingdom). The procedures of metagenomic sequencing are detailed in https://en.novogene.com/services/research-services/metagenomics/shotgun-metagenomic-sequencing/ and Zhang et al. (2018).

The gene catalogs have been depicted in Supplementary Table 1 and Figure 3. Taxonomy annotation analysis involved comparing metagenomic reads to the database of taxonomically informative gene families (NR database) to annotate each metagenomic homolog. Taxonomic diversity involves identifying those reads that are marker gene homologs to a database of taxonomically informative gene families using sequence or phylogenetic similarity to the database sequences (NR database) (Buchfink et al., 2015) to taxonomically annotate each metagenomic homolog (MEGAN, Huson et al., 2011).

**FIGURE 1:**
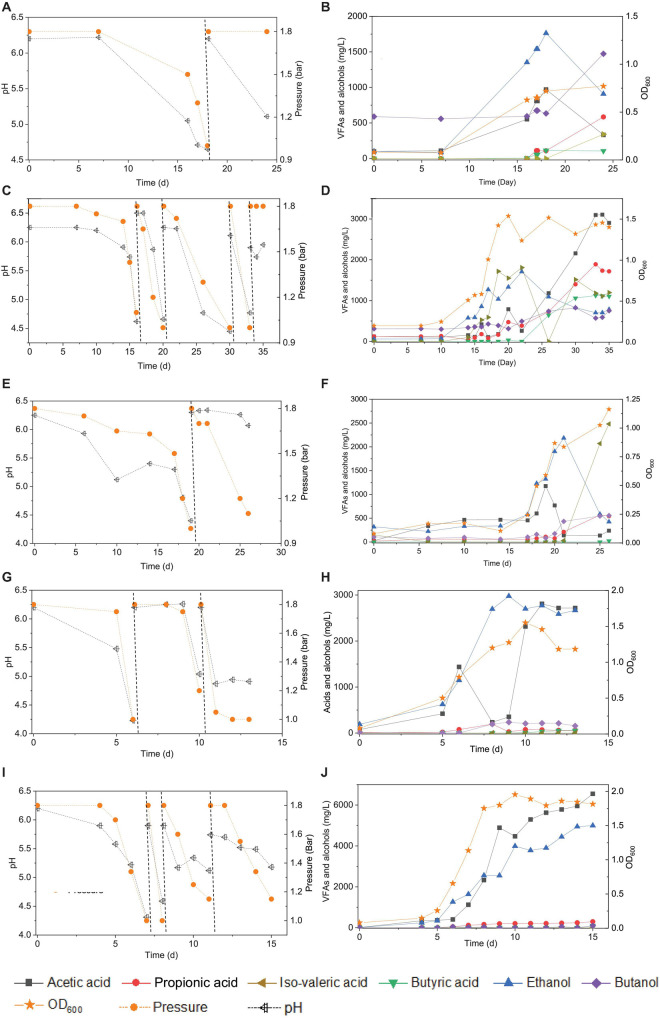
Enrichment of CO-converting solventogenic bacteria among the five transfers using CO as the carbon source by enriched sludge with initial CO gas pressure of 1.8 bar. Panels **(A,C,E,G,I)** represent the changes of gas pressure and pH of the 1st, 2nd, 3rd, 4th, and 5th transfers, respectively. Panels **(B,D,F,H,J)** represent cell concentration (OD_600_), acetic acid (HAc), propionic acid (HPr), butyric acid (HBu), ethanol (EtOH), isovaleric acid (i-Hval), and butanol (BtOH) production of the 1st, 2nd, 3rd, 4th, and 5th transfers, respectively. The dashed lines in panels **(A,C,E,G,I)** represent 1.8 bar CO addition and pH adjustment.

**FIGURE 2:**
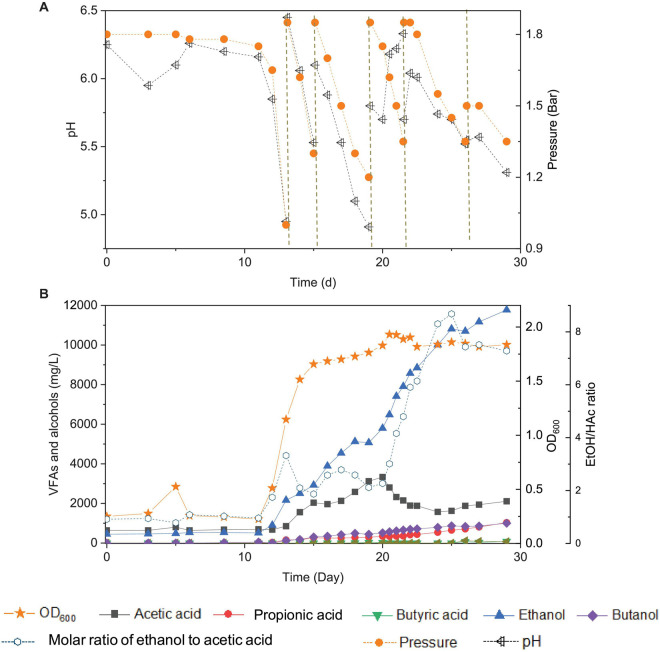
Changes of pH and gas pressure **(A)** and cell concentration (OD_600_), acetic acid, propionic acid, butyric acid, ethanol, butanol, and molar ratio of ethanol to acetic acid **(B)** using CO as the carbon source with initial CO gas pressure of 1.8 bar in the 6th transfer by enriched sludge. The dashed lines in panel **(A)** represent 1.8 bar CO addition and pH adjustment.

**FIGURE 3:**
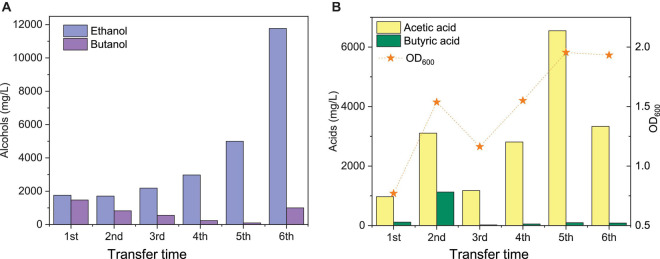
Comparison of the maximum ethanol and butanol concentrations **(A)** and acetic acid, butyric acid, and cell concentrations (OD_600_) **(B)** of the 1st, 2nd, 3rd, 4th, and 5th transfers using CO as the carbon source by enriched sludge.

According to the abundance table of each taxonomic level, various analyses were performed including Krona analysis, bar plot for abundant species, and heatmap of abundance. Principal component analysis (PCA) based on Bray–Curtis distance was used to evaluate the similarity of samples. The distance was calculated according to relative taxonomic abundance (Buchfink et al., 2015). The final results were exhibited by combining the clustering result and relative abundance of different samples at the phylum level.

## Results

### Enrichment of Carbon Monoxide-Converting Acetogens

In the 1st transfer, CO was added twice (i.e., two CO additions) at 0 and 18 d (Figure 1A). After 16 days of fermentation, 0.6 g/L acetic acid and 1.3 g/L ethanol were produced (Figure 1B). Both the acetic acid and ethanol concentrations reached their highest value of 0.9 and 1.7 g/L, respectively, at day 18 along with a gas pressure decrease from 1.8 to 1.0 bar, and the pH dropped to 4.65 (Figures 1A,B). Then, both the acetic acid and ethanol concentrations decreased to 0.5 and 1.0 g/L, respectively, at day 24. The OD_600_ gradually increased to a maximum value of 0.72 after 24 days of incubation. A 0.1 g/L butyric acid had accumulated at day 18 and was kept stable till the end of incubation at day 24, while butanol production was stimulated during the second CO fermentation and reached 1.0 g/L at day 24. Some other acids were detected as well, such as 0.6 g/L propionic acid and 0.3 g/L valeric acid, which further contributed to induce a pH decrease to 5.11 at the end of the incubation (Figure 1A).

In the 2nd transfer, CO was added at 0, 16, 21, 30, and 33 d, respectively (i.e., five CO additions) (Figure 1C). Ethanol started being produced and reached 0.9 g/L at day 16, while the acetic acid concentration remained low, with only 0.4 g/L being detected, and the pH dropped to 4.6 (Figure 1D). Then, the ethanol concentration kept increasing to 1.7 g/L, while the acetic acid concentration was 0.3 g/L along with the pH decreasing to 4.7 and the OD_600_ reaching its highest value of 1.537 at day 22 (Figure 1D). Thereafter, acetic acid started to accumulate quickly and reached 3.1 g/L, while the ethanol concentration decreased to 0.7 g/L at the end of the incubation (Figure 1D). Meanwhile, the net amounts of butyric acid and butanol increased to 1.1 and 0.8 g/L, respectively (Figure 1D). The concentrations of propionic and valeric acids were 1.7 and 1.2 g/L, respectively, at the end of the incubation (Figure 1D).

During the 3rd transfer, CO was supplied twice, at days 0 and 19. After approximately 17 days of adaptation, the net acetic acid and ethanol concentrations increased to reach 1.1 and 1.0 g/L, respectively, at day 19, with the pH dropping to 4.4 (Figures 1E,F). The net amount of ethanol reached its highest concentration of 1.8 g/L, along with a steep decrease of the acetic acid concentration to 0.1 g/L at day 22 when the gas pressure decreased from 1.8 to 1.7 bar (Figures 1E,F). However, the ethanol and acetic acid concentrations decreased to 0.4 g/L at day 26 (Figure 1E). The butanol concentration increase occurred after adding CO at pH 6.2 at day 19, and then it remained stable at 0.6 g/L (Figure 1F). Both 0.5 g/L propionic acid and 2.5 g/L valeric acid were also found at day 26 (Figures 1E,F), when the highest cell concentration of 1.163 (OD_600_) was obtained.

With the 4th transfer, CO was supplied at days 0, 6, and 10, respectively. The net ethanol and acetic acid production reached 1.0 and 1.4 g/L, respectively, along with the pH decreasing to 4.23 at day 6 (Figures 1G,H). The ethanol concentration kept increasing and reached 2.4 g/L, with the pH dropping to 5.0 (Figures 1G,H). Finally, the ethanol concentration slightly increased to 2.8 g/L at day 11 of the last CO addition (Figures 1G,H). The cell concentration increased to 0.793 after the first CO addition and reached the highest value of 1.55 after the second CO addition (Figures 1G,H).

Acetic acid and ethanol production were dominant in the 5th transfer but with a quite higher ethanol concentration than in all previous transfers (Figure 1I). CO was supplied four times at days 0, 7, 8, and 12 (Figure 1I). After 5 days of adaptation to the 5th transfer, the acetic acid and ethanol concentrations increased to 0.4 and 1.3 g/L, respectively, with the pH decreasing to 5.22, while the OD_600_ increased to 0.655 at day 7 (Figures 1I,J). The second CO addition lasted only 1 day, with a very fast gas consumption, and the acetic acid and ethanol concentrations increasing to 2.3 and 2.6 g/L, respectively, with the pH dropping to 4.31 and the OD_600_ increasing to 1.753 at day 8 (Figures 1I,J). With the last two CO additions in the 5th transfer, the cell concentration remained roughly stable, while acetic acid and ethanol concentrations increased to 6.5 and 5.0 g/L, respectively (Figures 1I,J).

The enrichment strategy with five successive transfers was efficiently selected for acetic acid/ethanol-producing organisms, with only minor concentrations of other metabolites, such as propionic and valeric acids. The unstable propionic acid and valeric acid production at the 1st, 2nd, and 3rd transfers could be attributed to the change of microbial community composition during the enrichment, for instance, the relative abundance of *Clostridium* bacteria was enriched up to 52% at the 2nd transfer, while it increased to 78% at the 4th transfer (Table 1). The exponential biomass growth phase led to an exponential production of metabolites, while the accumulation of end products slowed down once the steady biomass concentration was reached (Figure 1). Ethanol consumption, which was observed toward the end of each incubation period during the first three transfers, was not observed anymore from the fourth transfer onward (Figure 1). The net ethanol production was enhanced threefold at the 5th transfer compared with the first one. The relatively high production of 5.0 g/L ethanol in batch tests using CO as the carbon source was seldomly reported so far, except some studies conducted in bioreactors using syngas as the substrate (Table 2). This high ethanol concentration can be even significantly increased further, as shown in the additional experiments described hereafter.

**TABLE 1:** Relative abundance of *Clostridium* spp. at genus level in the initial inoculum sludge and the 2nd, 4th, 5th, 6th, 7th, and 8th transfers as well as in the incubation with exogenous butyric acid (HBu) and glucose addition (Glucose), controlled at pH 6.2 and 5.7.

	**Inoculum sludge**	**2nd transfer**	**4th transfer**	**5th transfer**	**6th transfer**	**7th transfer**	**8th transfer**	**HBu**	**Glucose**	**pH 6.2**	**pH 5.7**
**Inoculum source**								**5th transfer enriched sludge**	**6th transfer enriched sludge**	**7th transfer enriched sludge**	
The relative abundance of *Clostridium* genus in bacteria	7	52	78	94	81	84	88	88	9	81	88
The relative abundance of *Clostridium* genus in the total sample	4	43	66	85	73	73	81	82	7	69	76

**Species name**	***Clostridium* (genus level)%**										

*Clostridium* strain W14A	29	0.7	2	0.9	2	1	1	0.4	**11**	2	1
*Clostridium ragsdalei*	10	2	4	4	4	4	4	4	3	4	4
*Clostridium estertheticum*	5	0.008	0.007	0.009	0.0007	0.002	0.002	0.0007	0.05	0.005	0.01
*Clostridium ljungdahlii*	3	8	8	8	8	8	8	8	0.9	8	8
*Clostridium coskatii*	2	4	4	5	4	4	4	4	0.6	4	4
*Clostridium magnum*	1	0.3	0.3	0.1	0.06	0.08	0.3	0.1	4	0.4	0.5
*Clostridium autoethanogenum*	1	5	5	6	6	6	6	6	0.4	5	5
*Clostridium* sp. PI S10 A1B	0.9	0.2	0.07	0.01	0.01	0.07	0.02	0.05	2	0.1	0.03
*Clostridium amylolyticum*	0.8	0.2	0.09	0.03	0.05	0.04	0.04	0.03	2	0.08	0.06
*Clostridium botulinum*	0.7	0.2	0.3	0.2	0.2	0.2	0.2	0.2	2	0.2	0.2
*Clostridium homopropionicum*	0.4	0.02	0.04	0.01	0.004	0.003	0.1	0.01	**10**	0.05	0.03
*Clostridium carboxidivorans*	0.6	0.4	0.4	0.4	0.3	0.7	0.6	0.3	0.8	0.4	0.4
*Clostridium kluyveri*	0.6	0.3	0.4	0.3	0.3	0.3	0.4	0.3	0.4	0.3	0.3
*Clostridium tyrobutyricum*	0.5	0.7	0.8	0.8	0.7	0.7	0.8	0.7	0.2	0.7	0.7
*Clostridium* sp. C105KSO15	0.08	0.009	0.08	0.002	0.01	0.04	0.04	0.4	3	0.01	0.004
*Clostridium cadaveris*	0.07	0.5	0.7	0.1	0.2	0.2	0.2	1	**14**	0.5	0.4
*Clostridium butyricum*	0.07	0.009	0.02	0.005	0.0006	0.002	0.02	0.003	1	0.03	0.06
*Clostridium* sp. BNL1100	0.03	0.002	0.003	0.0001	0.0003	0.002	0.007	0.008	1	0.03	0.01
Other identified *Clostridium* spp.	17.3	6.5	2.8	1.1	1.2	2.7	1.3	1.5	14.7	2.2	3.3
**Unidentified *Clostridium* spp.**	**27**	**71**	**71**	**73**	**73**	**72**	**73**	**73**	**29**	**72**	**72**

*The bolded values in Glucose column represent the increased relative abundance of Clostridium spp. compared to the successive transfers. The bolded values in the last row represent the high relative abundance of unidentified *Clostridium* spp.*

**TABLE 2:** Highest ethanol and butanol concentrations during syngas/CO fermentation using various biocatalysts.

**Microorganism**	**Reactor configuration**	**Gas composition**	**Working volume (L)**	**Time/d**	**pH**	**Highest alcohols (g/L)**		**References**
						**Ethanol**	**Butanol**	
*Alkalibaculum bacchi* CP15	CSTR	CO/CO_2_/H_2_/N_2_ (20/15/5/60)	3.3/7	51	8.0	6.0	1.1	Liu et al., 2014
*C. carboxidivorans* P7	Bubble column	CO/CO_2_/N_2_ (25/15/60)	4.5/6.2	10	5.3–5.75	1.6	0.6	Rajagopalan et al., 2002
	HFR	CO/CO_2_/H_2_/N_2_ (20/15/5/60)	8	15	6	24.0	NA	Shen et al., 2014
	CSTR	CO/CO_2_/H_2_/N_2_ (20/15/5/60)	3/7.5	11	5.7	1.5	0.5	Ukpong et al., 2012
	CSTR	100% CO	1.2/2	21	5.75, 4.75	5.55	2.66	Fernández-Naveira et al., 2016
	Batch	CO/CO_2_/H_2_/Ar (56/20/9/15)	0.03/0.125	5	NA	3.64	1.35	Shen et al., 2020
*C. autoethanogenum*	CSTR	100% CO	1.2/2	7	6.0, 4.75	0.9	NA	Abubackar et al., 2015
	Batch	100% CO	0.075/0.2	NA	5.75, 4.75	0.65	NA	Abubackar et al., 2012
*C. ljungdahlii*	CSTR + Bobble column	CO/CO_2_/H_2_ (60/5/35)	1/2 (CSTR) 4/6 (BC)	83	5.5 (CSTR) 4.3–4.8 (BC)	20.7	NA	Richter et al., 2013
*Clostridium aceticum*	CSTR	CO/CO_2_/H_2_/N_2_ (30/5/15/50)	1.2/2	52	6.98	5.6	NA	Arslan et al., 2019
*C. ragsdalei*	Tricking bed reactor	CO/CO_2_/H_2_/N_2_ (38/28.5/28.5/5)	1	70	5.8–4.6	5.7	NA	Devarapalli et al., 2016
*Clostridium* strain P11	CSTR	CO/CO_2_/H_2_/N_2_ (20/15/5/60)	3.5/7.5	15	6.1	5.0	0.6	Maddipati et al., 2011
Anaerobic sludge from industry wastewater treatment	CSTR	100% CO	1.2/2	42	6.2, 4.9	11.1	1.8	Chakraborty et al., 2019
Enriched sludge in fed batch (6th transfer)	Fed batch	100% CO	0.1/0.5	29	5.0–6.3	11.8	1.0	This study

### Enhanced Ethanol Production With Minor Acetic Acid Accumulation

In the 6th transfer, CO-converting acetogens with enhanced ethanol production were successfully enriched compared with the previous five transfers. Six CO supplies were performed in the 6th transfer, at 0, 13, 15, 19, 21 and 26 d, with the initial CO gas pressure set at 1.8 bar (Figure 2A).

In the first CO addition (0–13 d), 11 days of adaptation was required, and then 0.8 g/L acetic acid and 2.2 g/L ethanol were produced, with an ethanol to acetic acid molar ratio of 3.32 (Figure 2B). This lag phase of several days, just after inoculation, was typically observed in all transfers indicating gradual adaptation to the conditions, while subsequent fast gas consumption occurred at each new CO supply. The pH value decreased to 4.95, and the gas pressure decreased to 1 bar at day 13 (Figure 2A). The second CO addition (13–15 d) lasted only 48 h as the gas pressure quickly dropped to 1.2 bar and the pH decreased to 5.5 (Figure 2A) and yielded 2.1 g/L acetic acid and 3.0 g/L ethanol. The molar ratio of ethanol to acetic acid decreased to 1.87, due to the high acetic acid accumulation in the biomass log phase (Figure 2B). The cell concentration increased to 1.685 (OD_600_) (Figure 2B).

At the third CO addition (15–19 d), the pH decreased to 4.91, and ethanol kept accumulating up to 5.1 g/L, while the acetic acid concentration only slowly increased to 3.1 g/L (Figure 2B). Around this period, biomass growth and the acetic acid concentration leveled off, while ethanol production kept increasing at high rates, except for a short stable period at day 18–19, corresponding to a gas pressure that decreased to 1.2 bar and a pH value that reached its lowest value (pH < 5) of the whole experiment (Figure 2). A similar slowing down in the ethanol production was observed at the end of the first CO addition from the 1st to 5th transfer, at low gas pressure and pH 4.95 (Figure 1). A minor increase in cell concentration was observed, up to 1.765 (OD_600_), at day 19. At the fourth CO addition (19–21 d), the acetic acid concentration decreased for the first time, along with the pH increasing from 5.7 to 6.18 at day 20 (Figure 2B). To sustain acetic acid consumption and minimize any potential ethanol oxidation sometimes reported in the presence of CO_2_ (Arslan et al., 2019; He et al., 2022), the pH was readjusted to 5.7. The accumulation of ethanol reached 7.9 g/L, while the acetic acid concentration decreased to 2.1 g/L, and the ethanol to acetic acid molar ratio reached 4.80 (Figure 2B). The highest cell concentration of 1.932 (OD_600_) was then measured at day 21, and cell growth entered a steady phase. The gas pressure decreased to 1.35 bar at day 21 (Figure 2A).

During the fifth CO addition (21–26 d) of the 6th transfer, ethanol was the only compound with increasing concentration, up to 10.7 g/L, with the highest ethanol/acetic acid molar ratio of 8.68 at day 25 (Figure 2B). A final, high ethanol concentration of 11.8 g/L was obtained with only 2.1 g/L acetic acid, 1.0 g/L propionic acid, and 1.0 g/L butanol and insignificant butyric acid production (0.07 g/L) at the end of the incubation (Figure 2B).

The highest net ethanol concentrations of the 1st, 2nd, 3rd, 4th, 5th, and 6th transfers were 1.7, 1.7, 1.9, 2.8, 5.0, and 11.8 g/L, respectively (Figure 3A). Conversely, the highest net butanol production during the six transfers was 0.9, 0.5, 0.5, 0.2, 0.1, and 1.0 g/L, respectively, from the first to the last transfer (Figure 3A). The ethanol concentration was enhanced 6.9-fold between the 1st and the 6th transfers. The butanol concentration at the 5th transfer reached only 1/9 of the value corresponding to the 1st transfer (Figure 3A). The net butyric acid production also decreased along with the enrichments, which reached its highest concentration of 0.2 g/L in the 1st transfer and 1.1 g/L in the 2nd transfer; then, it remained below 0.1 g/L at the 3rd, 4th, 5th, and 6th transfers (Figure 3B). Despite the low butanol production, interestingly, its concentration increased slowly, although butyric acid was not significantly produced after six enrichments (Figure 3A). The highest butanol concentration reached 1.0 g/L and with only 0.085 g/L butyric acid production at the end of the incubation period (Figures 3A,B).

**FIGURE 4:**
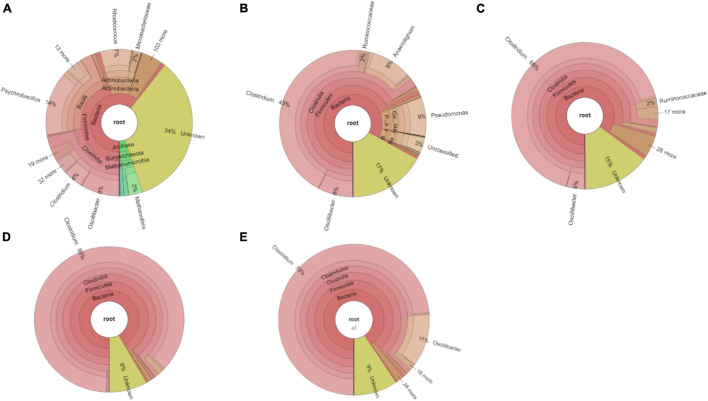
Relative abundance at genus level of **(A)** the initial inoculum, **(B)** 2nd, **(C)** 4th, **(D)** 5th, and **(E)** 6th transfers for enriched acetogens using CO as the carbon source.

The relative abundance of the initial inoculum was 61% bacteria, 5% archaea, and 34% unknown. The phylum Firmicutes occupied 75% of the bacteria, mainly represented by the Clostridia (47%) and Bacilli (49%) classes (Figure 4A). The Clostridiales order occupied 98% in the Clostridia class, which was mainly distributed over the Ruminococcaceae (14%), Clostridiaceae (21%), and Oscillospiraceae (40%) families (Figure 4A). After successive transfers and enrichments, the *Clostridium* genus increased from 7% in the inoculum sludge to 52, 78, 94, and 81%, respectively, in the 2nd, 4th, 5th, and 6th transfers of the enriched cultures (Figure 4 and Table 1).

Among the *Clostridium* genus, some identified species such as *C. autoethanogenum* increased from 1 to 6%, which is known to convert CO to acetic acid and ethanol *via* the WLP pathway (Tables 1, 2). The relative abundance of *C. ljungdahlii* increased from 3 to 8% after enrichment, which is also a species producing aetate and ethanol from syngas *via* the WLP (Mohammadi et al., 2012; Tables 1, 2). *C. carboxidivorans* produces butanol from CO (Fernández-Naveira et al., 2016), besides ethanol, but its relative abundance did not significantly increase and even slightly decreased to 0.4% in the 5th transfer (Tables 1, 2). Unidentified *Clostridium* species occupied a very high relative abundance, increasing from 27% in the inoculum to 71, 71, 74, and 73% of the *Clostridium* genus in the 2nd, 4th, 5th, and 6th transfers, respectively (Table 1). The 7th and 8th transfers were further conducted in successive transfers, and the relative abundance of the *Clostridium* genus occupied as high as 73 and 81%, respectively, similar to the 5th and 6th transfers (Supplementary Figures 1, 2).

**FIGURE 5:**
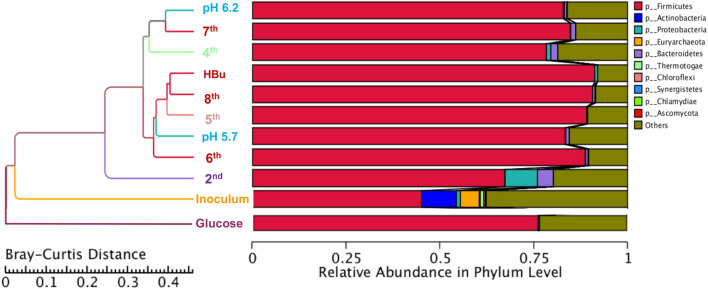
Clustering tree based on the Bray–Curtis distance of the inoculum, 2nd, 4th, 5th, 6th, 7th, 8th, and exogenous butyric acid addition.

**FIGURE 6:**
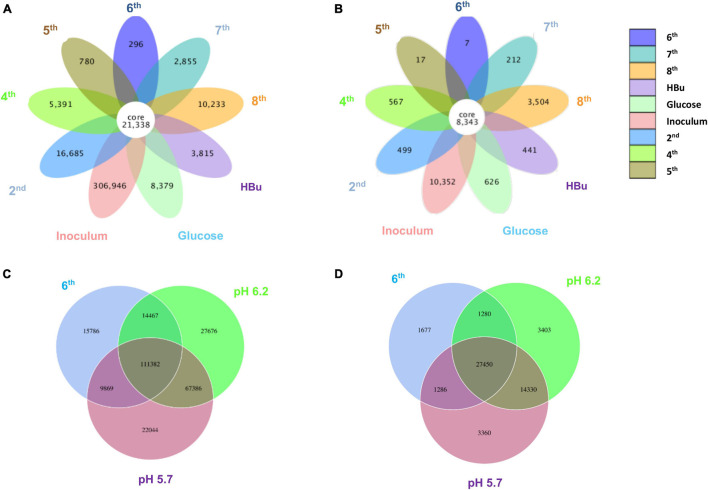
Venn diagrams of **(A)** the whole genes and **(B)** genes in *Clostridium* genus level present in the inoculum, 2nd, 4th, 5th, 6th, 7th, 8th, and exogenous butyric acid addition of enrichment study; **(C)** the whole genes and **(D)** genes in *Clostridium* genus level pH 5.7, pH 6.2, and 6th transfer enriched sludge for ethanol production under pH control. The overlapping parts represent the number of common genes among samples (groups); the other parts represent the number of special genes present in a particular sample.

Figure 5 shows a clustering tree based on the Bray–Curtis dissimilarity and the relative abundance at phylum level among the initial inoculum and the successive transfers. The initial inoculum had a high dissimilarity with the successive transfers, while the 2nd transfer had high dissimilarity with the subsequent transfers, which corresponds to the high differences in the relative abundance at genus level (Figure 5 and Table 1). The highest dissimilarity was observed between glucose fermentation (see Section “Glucose and CO Co-fermentation”) and the other assays. The common and special genes among the transfers are shown in the Venn diagrams (Figure 6). In the common core genome of 21,338, specific genes were decreased along with the successive transfers. For instance, the inoculum had the highest number of genes (306,946), while the 6th transfer had the least specific genes (296) (Figure 6A). Considering the high abundance of the *Clostridium* genus, a Venn figure for gene analysis at *Clostridium* genus level was analyzed. Interestingly, the 5th and 6th transfers contained only 17 and 7, respectively, special genes in the *Clostridium* genus (Figure 6B).

### Butanol Production From Exogenous Butyric Acid Using Carbon Monoxide as Reducing Power

**FIGURE 7:**
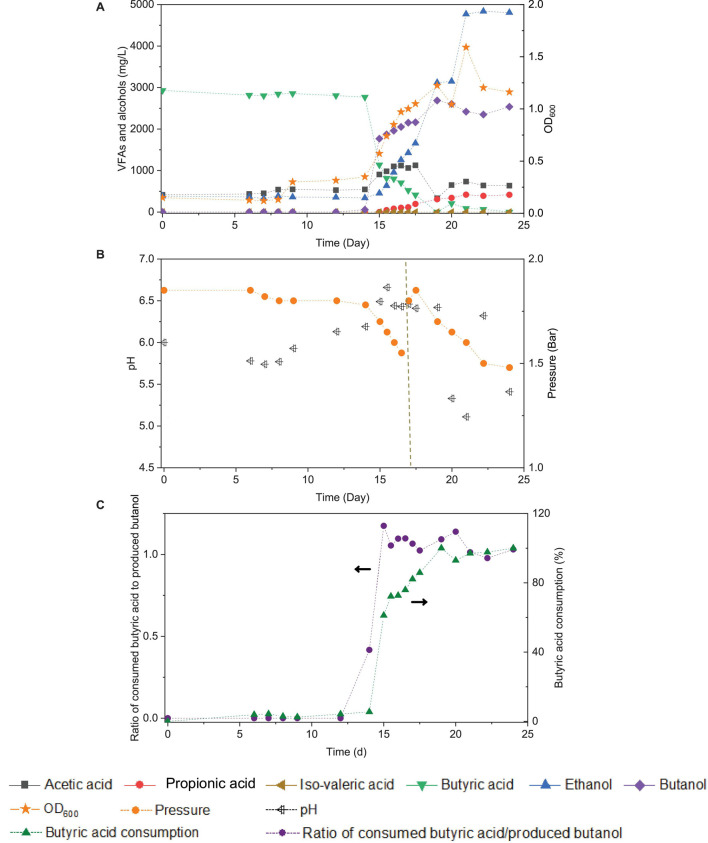
Effect of exogenous 3.2 g/L butyric acid on acetic acid, propionic acid, iso-valeric acid, butyric acid, ethanol and butanol production, and cell concentrations (OD_600_) **(A)**, change of pH and gas pressure **(B)**, and molar ratio of butyric acid consumption/butanol production and butyric acid consumption percentage **(C)** using CO as the carbon source with initial CO gas pressure of 1.8 bar by enriched sludge.

Upon the addition of exogenous butyric acid inoculated with the 5th transfer enriched sludge taken at the end of incubation and twice CO addition (days 0 and 16), the cell growth entered the log phase after 14 days of adaptation: fast and high butanol production was observed and reached 1.8 g/L in less than 1 day, while butyric acid decreased from its initial value of 3.2–1.1 g/L at day 15 (Figure 7A). Meanwhile, acetic acid and ethanol production reached 1.1 and 1.3 g/L, respectively, at the end of the first CO addition (day 16), suggesting simultaneous butanol production from exogenous butyric acid together with acetic acid and ethanol production from CO (Figure 7A).

Considering the possible ethanol consumption in the presence of accumulated CO_2_, and thus to avoid CO_2_ build-up, 1.8 bar CO was added in the headspace on day 17 (Figure 7B). The amount of butanol kept increasing, reaching its highest concentration of 2.7 g/L at the end of the incubation, while butyric acid was completely exhausted. Complete exogenous butyric acid consumption and the end of butanol production occurred simultaneously. At the end of the incubation period, ethanol had reached its highest concentration of 4.8 g/L, and acetic acid remained at a relatively low concentration of 0.6 g/L (Figure 7A). The pH was adjusted manually to 6.2 each time its value either exceeded 6.5 or decreased below 5.2. The production of butanol from butyric acid and CO was observed over the pH range 6.2- 6.5 (Figure 7B). When the pH exceeded 6.5 or was lower than 5.2 (20–24 d), butanol production did insignificantly increase (Figure 7B).

**FIGURE 8:**
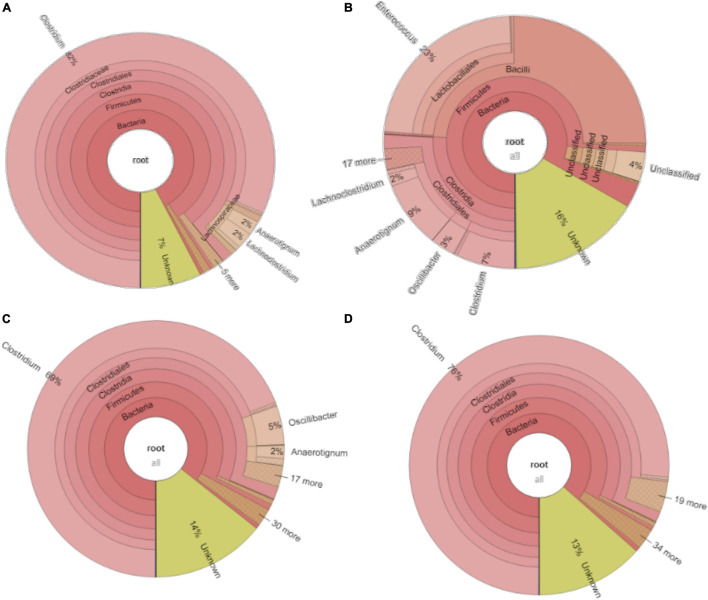
Microbial community analysis of **(A)** exogenous 3.2 g/L butyric acid addition inoculated with the 5th transfer enriched sludge, **(B)** exogenous 5 g/L glucose addition inoculated with 6th transfer enriched sludge; **(C)** pH 6.2 and **(D)** pH 5.7 inoculated with 7th enriched sludge.

For the enriched sludge after converting butyric acid to butanol, with 100% conversion efficiency, the relative abundance of the *Clostridium* genus increased from 73 to 82%, but the relative abundance at species level, such as *C. ljungdahlii*, *C. autoethanogenum*, *Clostridium ragsdalei*, and *Clostridium coskatii*, did not change much compared with the enriched sludge of the 6th transfer (Table 1). The genus *Oscillibacter* with 11% in the 6th transfer inoculum disappeared after exogenous butyric acid conversion, while the relative abundance of the *Anaerotignum* genus and *Lachnoclostridium* genus were both slightly increased to 2% in the sludge after converting exogenous butyric acid to butanol (Figure 8A).

### Glucose and Carbon Monoxide Co-fermentation

**FIGURE 9:**
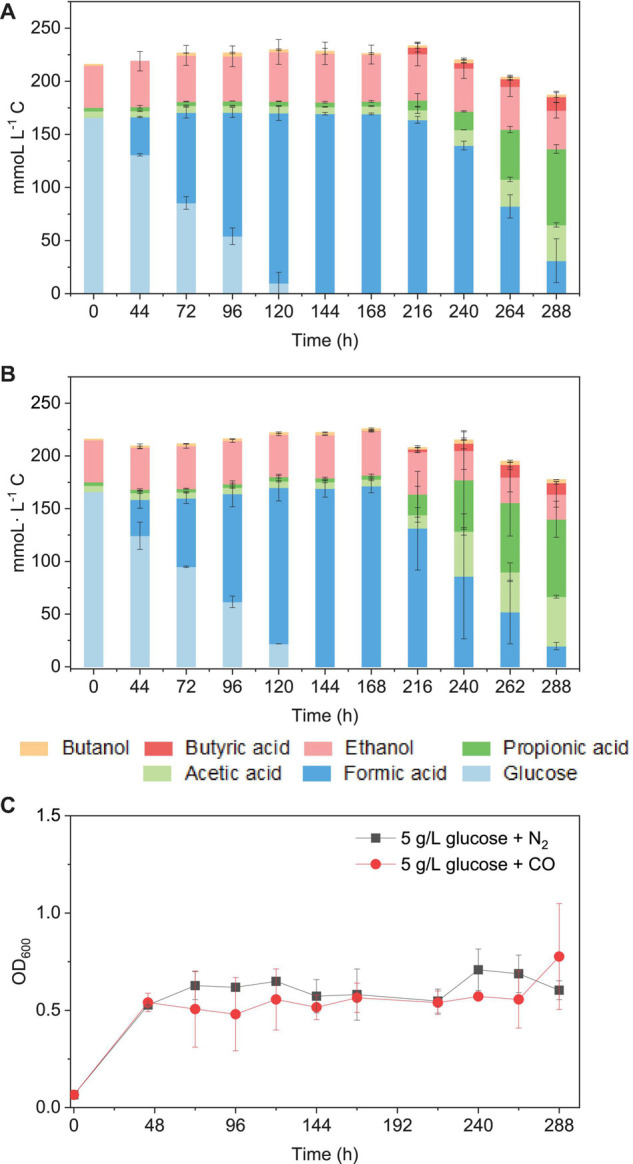
Glucose consumption and acetic acid, propionic acid, butyric acid, and ethanol and butanol production in mmoL⋅L^–1^ C using **(A)** 5 g/L glucose + N_2_ and **(B)** 5 g/L glucose + CO as the substrate by the enriched sludge (6th transfer) and **(C)** cell concentration (OD_600_).

When using 5 g/L glucose (166 mmoL⋅L^–1^ C) as the sole substrate (with N_2_ as the gas phase), it was totally consumed with production of formic acid at 120 h, and formic acid accumulation (163 mmoL⋅L^–1^ C) was observed at 216 h. Then, formic acid was further converted to acetic acid, followed by propionic acid (Figure 9A). Glucose was consumed in 120 h (Figures 7A,B), and the presence of CO did not influence much the glucose consumption rate compared with N_2_ as the gas phase. The mmoL⋅L^–1^ carbon balance remained relatively stable during glucose consumption and formic acid production, although a small part of carbon could be used for cell growth. When glucose was completely consumed, 10–15% carbon loss was observed in the total mmoL⋅L^–1^ carbon after 240 h, which was possibly due to the carbon lost as CO_2_ during solventogenesis (Eq. 1) (Figures 9A,B). Biomass growth was very similar in both cases and reached an OD_600_ of approximately 0.5 at 44 h and then later the highest OD_600_ of 0.71 and 1.08, respectively, in the incubation with solely glucose or glucose and CO (Figure 9C). The enriched acetogens can thus also use glucose as the carbon source, which results in the accumulation of formic acid, followed by the slow production of acetic acid and propionic acid (Figure 9).


(1) C4⁢H7⁢O2-+H++CO→C4⁢H10⁢O+CO2 Δ⁢G0=-36.5⁢kJ⋅moL-1

After glucose bioconversion, the microbial community significantly changed: the Clostridia class occupied only 26%, while the Bacilli class occupied as high as 49% of the total. In the Clostridia class, the genus *Anaerotignum* occupied 9%, and the *Oscillibacter* genus occupied 3%, while the *Clostridium* genus was remarkably decreased from 73% in the 6th transfer to 7% after glucose bioconversion (Figures 4E, 8B). The increased relative abundance of known *Clostridium* spp. compared with the 6th transfer inoculum was, respectively, *Clostridium* strain W14A from 2 to 11%, *Clostridium homopropionicum* from 0.004 to 10%, and *Clostridium cadaveris* from 0.2 to 14% (Table 1). The relative abundance of *Clostridium butyricum* and *Clostridium* sp. BNL1100 both increased from 0.002 to 1% compared with the 6th transfer inoculum (Table 1). On the other side, the abundance of *C. ljungdahlii*, *C. autoethanogenum*, and *C. coskatii* decreased below 1% (Table 1). In the Bacilli class, the genus *Enterococcus* occupied 47 with 27% of *Enterococcus faecalis* and unidentified Bacilli occupying 52% (Figure 8B).

### Carbon Monoxide Conversion With pH Controlled at 5.7 and 6.2

**FIGURE 10:**
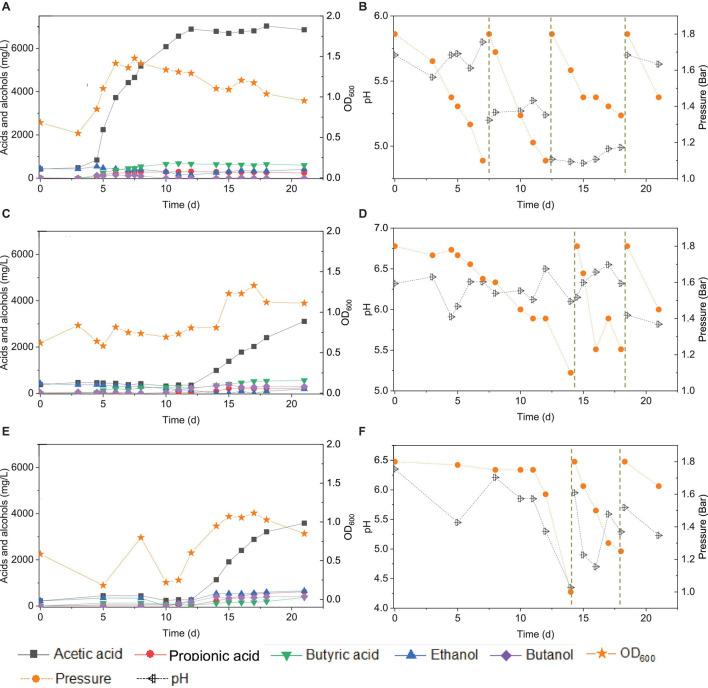
Acetic acid, propionic acid, butyric acid, ethanol and butanol production, and change of gas pressure and pH, respectively, under pH control of 5.7 **(A,B)**, 6.2 **(C,D)**, and manually pH adjustment **(E,F)** using CO as the carbon source with initial CO gas pressure 1.8 bar by the enriched sludge from the 7th transfer.

At pH controlled at 5.7 using the 7th transfer enriched sludge as the inoculum, the incubation entered the log phase on day 7 and reached an OD_600_ of 1.478 during the first CO addition (0–7 d) (Figure 10A). The acetic acid concentration increased to 4.6 g/L, while no increase in ethanol concentration was observed, and the butyric acid concentration reached 0.45 g/L at day 7. Considering the unfavorable ethanol production but high acetic acid accumulation, the pH was adjusted to 5.2 in order to try to stimulate solvent production at the second CO addition on day 7. However, the acetic acid concentration kept increasing and reached its highest concentration of 6.9 g/L at day 12. The butyric acid concentration increased to 0.66 g/L. The pH was adjusted to 4.9 at the third CO addition at day 12. Both acetic acid and the cell concentrations remained stable until the end of the incubation. The pH was adjusted back to 5.7 at the fourth CO addition, but it did not enhance acetic acid or ethanol production (Figures 10A,B).

At pH controlled at 6.2, a longer adaptation time of 12 days was observed than at pH 5.7 (Figure 10C). The acetic acid concentration increased to 0.99, while 0.3 g/L ethanol, carried over from the inoculum, was completely consumed by day 14. The butyric acid and butanol concentrations increased both to 0.3 g/L during the first CO addition (0–14 d) (Figure 10C). The second CO addition occurred at 14–18 d (Figure 10D). The concentrations of acetic, propionic, and butyric acids increased to 2.4, 0.2, and 0.5 g/L, respectively, while ethanol was not produced, and butanol remained stable at 0.3 g/L (Figure 9). The highest cell concentration reached an OD_600_ of 1.23 (Figure 10C). In the last CO addition (18–21 d), the acetic acid concentration increased to 3.1, while 0.2 g/L ethanol was produced.

The control incubation with initial pH 6.2 showed the same 12 days of adaptation time as the incubations with the pH controlled at 6.2. Three CO additions were applied at 0, 15, and 18 d. During the first CO addition (0–14 d), acetic acid production increased to 1.1 g/L along with a pH decrease to 4.35 and gas pressure decrease to 1 bar (Figures 10E,F). Ethanol increased to 0.5 g/L and butanol to 0.4 g/L, and the butyric acid concentration was 0.1 g/L. In the second CO addition (14–18 d), the acetic acid and ethanol concentrations increased to 3.2 and 0.6 g/L, respectively, along with the pH decreasing to 5.29. The propionic acid concentration reached 0.6 g/L. The highest cell concentration reached an OD_600_ of 1.114 at day 17. In the third CO addition, the acetic acid and ethanol concentrations slightly increased to 3.6 and 0.7 g/L, respectively (Figure 10E).

For the enriched sludge, at pH controlled at 6.2 or 5.7 using the 7th transfer as inoculum, in the family level, the relative abundance of Clostridiaceae, Oscillospiraceae, and Lachnospiraceae occupied 70%, 5% and 3%, respectively, of the total sample at pH 6.2, while the values were 77, 1, and 0.6%, respectively, at pH 5.7, compared with the inoculum in which they reached 74, 10, and 0.4%, respectively (Figures 8C,D). The relative abundance of the *Clostridium* genus occupied the highest value, with 69 and 76%, respectively, at pH 6.2 and 5.7, which are both close to the initial inoculum value of 73% (Figures 8C,D). The relative abundance of identified *Clostridium* spp. did not significantly change (Table 1). The differences among the *Clostridium* spp. in gene number and similarity among samples are shown in a Venn diagram (Figures 6C,D). Compared with the inoculum, the special gene numbers increased from 1,677 to 3,403, and 3,360, respectively, at pH 6.2 and 5.7 (Figure 6D).

## Discussion

### Enhanced Ethanol Production With Minor Accumulation of Acetic Acid by Highly Enriched Clostridium Sludge

This study showed that enhanced ethanol production was achieved with intermittent gas feeding and reached a maximum concentration of 11.8 g/L, with a high ethanol/acetic acid molar ratio of 8.68 in the 6th transfer (Figure 2B). The ethanol/acetic acid molar ratio was higher than 1 during the whole incubation and kept increasing after the adaptation stage (Figure 2B). It should be noted that such high ethanol production was, to the best of our knowledge, never reported from 100% CO bioconversion under a wide pH range from 4.95 to 6.45 (Table 2). Lee et al. (2019) isolated a novel strain *Clostridium* sp. AWRP from a wetland using syngas as the substrate. This strain produced 5.4 g/L ethanol and 0.9 g/L butanol in a batch bioreactor. Abubackar et al. (2015) compared pH 6 and 4.75 on ethanol production from CO in a CSTR by *C. autoethanogenum* and found that pH 4.75 favored ethanol production together with a negligible acetic acid concentration (<50 mg/L), but with the highest ethanol concentration of 0.867 g/L, which is quite lower than that obtained in the present research.

The present study shows that enhanced ethanol accumulation was achieved under a wide pH range from 6.45 to 4.95 (Figure 2A), and it can be concluded that the pH decreases induced a metabolic shift from acetogenesis to solventogenesis and allowed stepwise ethanol increases with low acetic acid accumulation, which may provide an efficient strategy for enhanced ethanol production. Moreover, the natural pH decrease could reduce ethanol oxidation in the presence of CO_2_ accumulation (Arslan et al., 2019) and thus further induce more ethanol production (He et al., 2022).

Ethanol production occurred both in the biomass log phase (11-19 d), reaching 5.1 g/L, and in the stable phase (19–29 d) of cell growth, with 11.8 g/L (Figure 2B). The increase in ethanol concentration in the steady growth phase was approximately 1.3-fold higher than that in the log phase. The decrease in acetic acid concentration was first observed when cell growth entered its stable phase at day 19 (Figure 2B). It is commonly assumed that solventogenesis occurs and is mainly promoted under non-growth conditions of homoacetogens (Mohammadi et al., 2011). For instance, *C. ljungdahlii* produces acetic acid and adenosine triphosphate (ATP) in the growth stage, and ethanol production generally occurs during the non-growth stage from CO, H_2_, and CO_2_ in the pH range of 4.0–7.0 *via* the acetyl-CoA pathway (Abubackar et al., 2011). However, this is not necessarily always the case. Cotter et al. (2009) observed little to no ethanol production in non-growing *C. ljungdahlii* cultures in a nitrogen-deficient medium. Besides, alcohol production during the biomass growth stage has also been observed (Figure 2B; Fernández-Naveira et al., 2017a).

From the genes analysis at *Clostridium* genus level, of the 8,343 shared genes present in the inoculum, the special genes in the 5th transfer amounted to 0.20% (17 special genes), and the 6th transfer only occupied 0.08% (7 special genes) (Figure 6B). Therefore, such high ethanol production with low acetic acid accumulation in the 6th transfer may not be related too much with the difference in *Clostridium* species with the 5th transfer, although both contain a high relative abundance of unidentified *Clostridium* (Table 1). Instead, the natural pH drop along with acetic acid accumulation triggers solventogenesis, and the timely pH adjustment at each CO addition prevented inhibition of biomass growth that can be caused by a further pH drop and thus allowed solventogenesis to prevail continuously. On the other hand, the stability of the microbial community was also demonstrated during the 4th, 5th, and 6th transfers with high amount of *Clostridium* genus.

Compared with the previous six transfers, neither ethanol nor butanol production was enhanced when this enrichment was incubated at a pH controlled at 5.7 or 6.2. The inhibition of ethanol production was first suspected to be related to the changes in microbial populations (Supplementary Figure 1). However, the relative abundance of the *Clostridium* genus was hardly affected in the assay at pH 5.7 compared with the inoculum; instead, it even showed a slight increase in *Clostridium* genus from 69 to 73% (Figure 8). The relative abundance of the Oscillospiraceae family decreased at both pH 6.2 and 5.7 compared with the inoculum, while the Lachnospiraceae family slightly increased. Esquivel-Elizondo et al. (2017) enriched Oscillospiraceae from CO or CO and H_2_ for mainly acetate production by anaerobic sludge. Considering the high abundance of unidentified *Clostridium* (Table 1), the failure of ethanol production might be due to differences of the unidentified *Clostridium* spp. among the enriched transfers at pH 6.2 and 5.7.

### Enrichment of Carbon Monoxide-Converting Microorganisms for Enhanced Ethanol Production

This study showed that the successive transfer procedure exerts a strong selective pressure on the microbial populations, with extreme enrichment of the *Clostridium* genus to end up representing >90% of the whole microbial community (Table 1). This resulted in a significantly higher ethanol accumulation, though butyric acid and butanol production became marginal, enriching thus for highly specific and efficient ethanol producers. To the best of our knowledge, to date, this paper is the first report providing such extensive microbial community analysis related to solventogenic CO fermentation, describing such a high amount unidentified *Clostridium* species enriched on CO (1.8 bar) from an anaerobic sludge. Chakraborty et al. (2019) conducted enrichments on CO and syngas for ethanol production from anaerobic sludge in a CSTR, and the species *C. autoethanogenum* was selectively enriched at pH 4.9 after 42 days of fermentation. Esquivel-Elizondo et al. (2017) investigated microbial community changes under different CO gas partial pressures (0, 8.1, 18.2, 40.5, and 81.1 kPa) and enriched mainly Eubacteriaceae, Ruminococcaceae, *Oscillospira*, and Bacteroidales with acetate as the main end product.

Solventogenesis can be stimulated to obtain a high ethanol production at the non-growth phase in *C. ljungdahlii* (Mohammadi et al., 2012). In this study, ethanol was produced, and its concentration increased in the log phase along with cell growth (Figure 9), which could be attributed to the high CO partial pressure (*P*_*CO*_, 1.8 bar) used in these experiments. Other authors also observed simultaneous alcohol production and biomass growth in species such as *C. autoethanogenum* (Abubackar et al., 2015) and *C. carboxidivorans* (Fernández-Naveira et al., 2017c) grown on C_1_ gases in bioreactors. Hurst and Randy (2010) reported that ethanol production was initiated at earlier times when increasing *P*_CO_ (from 0.35 to 2.0 bar), i.e., high *P*_CO_ changed ethanol production from non-growth-associated to growth-associated in *C. carboxidivorans* P7^T^.

*Clostridium* spp. were dramatically enriched with a majority of unidentified *Clostridium* species after successive transfers using CO with initial pressure of 1.8 bar as the carbon source and electron donor. The 4th, 5th, and 6th transfers showed less difference compared with the 2nd one, which corresponds to the enhancement of ethanol production after the 4th transfer. For instance, the relative abundance of the Lachnospiraceae family, medium chain fatty acid producers such as propionic acid, decreased from 11% in the 2nd transfer to below 1% after the 4th transfer (Figure 7). No significant clustering was observed from the 4th to the 8th transfer demonstrating the stability of the enriched solvent producing acetogens, i.e., *Clostridium* spp. using CO as the substrate. However, no significant change of known *Clostridium* spp. was observed in the 4th, 5th, and 6th transfers although high ethanol production was observed in the 6th enrichment transfer with low accumulation of acetic acid throughout the whole fermentation process (Table 1). On the other hand, the mixed culture composed of identified and unidentified *Clostridium* spp. could withstand pH changes and showed efficient solventogenesis over a wide pH range, which also explained the high ethanol production during 5th and 6th transfer co-feedings. Therefore, pH fluctuations in a mixed culture of *Clostridium* spp. may result in high, enhanced ethanol production, in which a high amount of unidentified strains may play a relevant role, concomitant with an only minor amount of residual acetic acid and a high ethanol/acetic acid ratio.

Nevertheless, the unfavorable butanol production observed here is likely due to unfavorable conditions, such as a low butyric acid production (precursor of butanol production), rather than the loss of butanol-producing acetogens considering the similar relative abundance of both *Clostridium* genus (81%) and *Clostridium* species in the incubations with exogenous butyric acid (88%) (Table 1). CO can be converted to acetic acid and ethanol *via* the acetyl-CoA by different enzymes of the WLP, and then acetyl-CoA can be further enzymatically transformed into butyryl-CoA from which butyric acid and butanol are produced (Fernández-Naveira et al., 2017c). Acid production in the acetogenic stage is necessary for alcohol production in the solventogenic stage (Worden et al., 1991). Butyric acid (C_4_) production occurs from the acetic acid (C_2_) carbon chain, which generally requires a higher pH (above 5.7) than solventogenesis (4.5–5.0) (Ganigué et al., 2016; He et al., 2022). Therefore, despite the diversity of the microbial community, the differences in butanol production among the different enrichments might be due to the different extents of pH fluctuation (Figures 1, 2). Environmental conditions may play a key role in different production profiles of metabolites, besides changes in microbial populations. Thus, it is suspected that the low butanol production among the transfers may be due to the low butyric acid concentration, which was possibly partly induced by unstable and unfavorable pH conditions.

### Enhanced Butanol Production From Exogenous Butyric Acid by Enriched Clostridium Populations

This study showed that the addition of exogenous butyric acid stimulated butanol production by the enriched culture with nearly 100% conversion efficiency in the assay, although butanol production was inhibited during the successive transfers (Figures 1, 2) using the same inoculum. This further demonstrated that the inhibition of butanol production during the enrichment process is possibly attributed to the lack of suitable conditions, such as the absence of butyric acid production. On the other hand, the failure of natural butyric acid production was most probably induced by the excessive pH drop under uncontrolled experimental pH conditions, such as the natural pH drop from 6.2 to 4.95 among the transfers (Figure 2). Thus, the pH value is considered to play an important role for obtaining butanol. Previous assays using the same enriched sludge yielded first 1.3 g/L butyric acid at controlled pH 6.2, with the accumulation of 2.1 g/L butanol when the pH was adjusted to 5.7 (He et al., 2022).

The molar ratio of butyric acid consumption to butanol production was close to 1, which matched with the theoretical molar ratio (Eq. 1) after 15 d (Figure 7C), and the butyric acid to butanol conversion efficiency reached almost 100%. Therefore, two metabolic reactions can be assumed to co-exist in the fermentation process: one being butanol production from exogenous butyric acid and CO (Liu et al., 2020) and the other being acetic acid production from CO with ethanol production *via* the WLP (Fernández-Naveira et al., 2017c). Interestingly, the molar ratio of ethanol/acetic acid reached 9.86 at the end of the incubation (Figure 7C), which is much higher than in other commonly reported studies of ethanol production from CO (Table 2).

From a microbial community point of view, the relative abundance of one of the only known butanol-producing species, *C. carboxidivorans*, was below 1% of the *Clostridium* genus in all the samples, which pointed to another possibility that the non-butanol-producing *Clostridium* strain, such as *C. autoethanogenum*, may change their metabolic pathway and produce butanol in the presence of butyric acid in a mixed culture (Diender et al., 2016). In the *Clostridium* genus level, the special genes of the enriched sludge after converting exogenous butyric acid to butanol (441 special genes) were much higher than those present in the inoculum (7 special genes), i.e., biomass from the 6th transfer (Figure 6B). The known *Clostridium* spp. were similar with the inoculum, and the extra special genes in the enriched sludge after converting butyric acid to butanol might be due to the difference in unidentified *Clostridium*. Therefore, it was suspected that the numerous unidentified *Clostridium* spp. might explain the different behaviors among samples considering that they exceeded 70% relative abundance in the *Clostridium* genus among the transfers (Table 1). The special genes of the enriched acetogens with exogenous butyric acid amounted to 441 and shared 95.0% of the genes with the biomass of the 6th transfer (8,343 common genes) (Figure 6B).

### Bioconversion of Glucose or Glucose and Carbon Monoxide Co-fermentation by the Enriched Culture

It has been reported that somewhat different metabolites may be obtained, even for a same bacterial pure culture, when either grown autotrophically on C_1_ gases or heterotrophically on sugars, e.g., glucose (Fernández-Naveira et al., 2017a, b). Formic acid accumulation was observed in both the glucose and the glucose and CO co-fermentation processes (Figure 9). Similarly, significantly early accumulation of formic acid from glucose was also observed in pure cultures of *C. carboxidivorans* (Fernández-Naveira et al., 2017b), while this was not found when that strain was grown on C_1_ gases (Fernández-Naveira et al., 2017a).

From the microbial community point of view, after glucose fermentation, the relative abundance of the *Clostridium* genus remarkably decreased to less than 1/10 compared with the inoculum within 12 days (288 h) of incubation (Figures 8, 9). *E. faecalis*, with increased relative abundance, can produce short chain fatty acids such as acetic acid using glucose as the substrate (Urdaneta et al., 1995). Despite the decrease in *Clostridium* genus, the relative abundance of *Clostridium* spp. varied at species level, with an increase in the abundance of *Clostridium* strain W14A, *C. homopropionicum* and *C. cadaveris* (Table 1). *Clostridium* strain W14A was isolated from a cellulose degrading biofilm in a landfill leachate microcosm (Ransom-Jones and Mcdonald, 2016). Similarly, with glucose as the substrate, *Clostridium* strain W14A was enriched reaching as high as 11% of the *Clostridium* genus in this study (Table 1). With sugar substrates, such as fructose, *C. homopropionicum* was observed to produce acetate, butyrate, butanol, and H_2_ (Dörner and Schink, 1990).

## Conclusion

This study describes a possible suitable strategy to enrich homoacetogens from anaerobic sludge for solventogenic ethanol and butanol production using CO as the carbon source after successive transfers and fed batch CO addition. The enriched acetogens with high *Clostridium* spp. abundance produced as much as 11.7 g/L ethanol with low accumulation of acetic acid over a wide pH range of 6.45–4.95. This selective ethanol production has been seldomly reported, especially with the low acetic acid accumulation under the pH range in the mixed culture. Besides, the enriched acetogens in the present study produced 2.7 g/L butanol from exogenous butyric acid with 100% conversion efficiency using CO as reducing power. Six successive transfers successfully enriched the *Clostridium* genus increasing from 7% in the inoculum to 94% in the solventogenic enrichment, including several well-known alcohol producers such as *C. ljungdahlii*, *C. autoethanogenum*, and *C. coskatii*, but with also unidentified *Clostridium* species occupying as high as 74% of the *Clostridium* genus.

## Data Availability Statement

The original contributions presented in the study are included in the article/Supplementary Material, further inquiries can be directed to the corresponding author/s.

## Author Contributions

YH carried out all experimental incubations and data analysis and drafted the manuscript. PL conducted the project supervision and the manuscript revision. MV provided the research resources. CK conceived the study, participated in its design and coordination, and reviewed the manuscript. All authors contributed to the article and approved the submitted version.

## Conflict of Interest

The authors declare that the research was conducted in the absence of any commercial or financial relationships that could be construed as a potential conflict of interest.

## Publisher’s Note

All claims expressed in this article are solely those of the authors and do not necessarily represent those of their affiliated organizations, or those of the publisher, the editors and the reviewers. Any product that may be evaluated in this article, or claim that may be made by its manufacturer, is not guaranteed or endorsed by the publisher.
