# Supplementary material for: Enhanced Ethanol Production From Carbon Monoxide by Enriched Clostridium Bacteria
Source: Front Microbiol. 2021 Oct 28;12:754713. doi: 10.3389/fmicb.2021.754713 (PMC8585497; doi:10.3389/fmicb.2021.754713)
Supplement: Supplementary file 1 [file Data_Sheet_1.docx]

Supplementary Material

**Enhanced ethanol production from carbon monoxide by enriched *Clostridium* bacteria**

**Yaxue He^1, 2^, Piet N.L. Lens^2^, María C. Veiga^1^, Christian Kennes^2*^**

^1^ Chemical Engineering Laboratory, Faculty of Sciences and Center for Advanced Scientific Research (CICA), BIOENGIN group, University of La Coruña (UDC), E-15008-La Coruña, Spain

^2^ National University of Ireland Galway, H91 TK33, Galway, Ireland

***Correspondence:**Christian Kennes
[c.kennes@udc.es](mailto:c.kennes@udc.es)

# Supplementary figures

**Supplementary Figure 1** Production of acetic acid (Hac), propionic acid (HPr), butyric acid (Hbu), ethanol (EtOH), isovaleric acid (i-Hval) and butanol (BtOH) of different transfer times and change of gas pressure and pH at 7^th^ using CO as the carbon source by enriched sludge with initial CO .

**Supplementary Figure 2** Relative abundance at genus level of the a) 7^th^ and b) 8^th^ transfer to enrich CO converting acetogens.


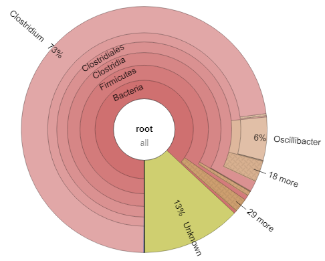


a)


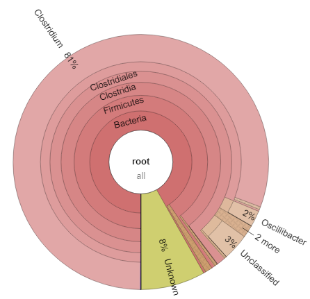


b)

**Supplementary Figure 3** Distribution of scaftig length (>=500bp)

(YH1- The enriched sludge at the end of the bioreactor for 6.8 g/L butanol production, YH2- 2^nd^ transfer, YH3- 4^th^ transfer, YH4- 5^th^ transfer, ER1- 6^th^ transfer, ER2- 7^th^, ER3- 8^th^, ER4- 5 HBu (Exogenous 3.2 g/L butyric acid), ER5- 6^th^ transfer grows on 5 g/L glucose, C1- pH 6.2, C2- pH 5.7).

**Supplementary Table 1** The Statistic of gene catalogues

| ORFs NO. | 954,675 |
| --- | --- |
| integrity: start | 150,041(15.72%) |
| integrity: none | 39,733(4.16%) |
| integrity: all | 547,831(57.38%) |
| integrity: end | 217,070(22.74%) |
| Total Len.(Mbp) | 590.04 |
| Average Len. (bp) | 618.06 |
| GC percent | 48.03 |

Note: "ORFs NO." means number of genes in gene catalogue. "integrity :start" represents amount and percentage of genes only containing start codon. "integrity: end" represents amount and percentage of genes only containing stop codon. "integrity: none" represents amount and percentage of genes not containing start or stop codon. "integrity: all" represents amount and percentage of genes containing both start and stop codon. "Total Len. (Mbp)" means the total length of gene catalogue (million). "Average Len." means the average length of genes in gene catalogue. "GC Percent" means the prediction of GC content of genes in gene catalogue.
